# Serum sHLA-G: Significant diagnostic biomarker with respect to therapy and immunosuppressive mediators in Head and Neck Squamous Cell Carcinoma

**DOI:** 10.1038/s41598-020-60811-y

**Published:** 2020-03-02

**Authors:** Vertica Agnihotri, Abhishek Gupta, Lalit Kumar, Sharmistha Dey

**Affiliations:** 1 Department of Biophysics, All India Institute of Medical Sciences, Ansari Nagar, New Delhi, India; 2 Medical Oncology, All India Institute of Medical Sciences, Ansari Nagar, New Delhi, India

**Keywords:** Oral cancer detection, Tumour biomarkers

## Abstract

Head & Neck Squamous Cell Carcinoma is one of the highest mortality factors in the world due to the lack of potential biomarker for early detection of disease. There is an urgent need for molecular marker involved in disease progression which remains suppressed normally, required for specificity. HLA-G is highly expressed in cancers and creates immune-suppressive microenvironment. Cancerous cells secrete inflammatory cytokines like IL-10,IFN-γ which increase expression of immunosuppressive molecules, such as HLA-G. We evaluated sHLA-G protein level in serum of 120 HNSCC patients at diagnosis and after therapy and compared with 99 individuals by SPR, ELISA and determined its mRNA level by qRT-PCR. sHLA-G was correlated with serum IL-10 and IFN-γ of the patients. Significant elevated levels of sHLA-G were observed in patients (8.25 ± 1.74 ng/µl) than control (6.45 ± 1.31 ng/µl). Levels were declined in (8.09 ± 1.79 ng/µl to 6.64 ± 1.33 ng/µl) patients in response to therapy. sHLA-G levels with tumor burden (8.16 ± 1.91 to 6.63 ± 1.32 ng/µl), node (8.62 ± 1.45 to 6.66 ± 1.26 ng/µl), PDSCC (8.14 ± 0.62 to 5.65 ± 0.27 ng/µl) and oropharynx (7.90 ± 1.24 to 6.10 ± 1.33 ng/µl) showed a positive and significant response to therapy. Findings indicate that sHLA-G can be a potential diagnostic serum protein marker for HNSCC due to its suppressive function and over expression in diseased condition with the influence of cytokines.

## Introduction

HLA-G is a Human Leukocyte Antigen G, restrictively expressed with suppressive functions^1^. Head and Neck Squamous Cell Carcinoma (HNSCC) is strongly influenced by environmental carcinogens (tobacco, alcohol etc.) which linked to loss of immune system to fight against cancer^2^. It is well established that HLA-G is highly expressed in many cancers and related to immune suppressive microenvironment^3^. Previous study on Chinese population reported correlation of serum sHLA-G with polymorphism in oral squamous cell carcinoma (OSCC)^4^. The cancerous cells secrete various inflammatory cytokines like IL-10, IFN-γ which increase expression of immunosuppressive molecules, such as HLA-G^5–7^. The expression of HLA-G also increases due to polymorphism at 3′UTR + *3142 G/C* genotype which restrict the binding of miRNA with mRNA in presence of C allele^8^. In our previous study, it has been reported that C/C genotype and C allele are major risk factors with strong contact of tobacco in North Indian HNSCC patients^9^. To further extend our previous work, this study has been performed to explore the effect of the presence of C/C allele on protein expression in serum of HNSCC patients which could be a simple and resourceful way for the development of serum based protein marker for the early diagnosis of the disease. Hence, the present study further correlated this polymorphism with serum levels of sHLA-G protein in HNSCC patients. This study first time evaluated the level of sHLA-G protein in serum of HNSCC patients and correlated with immunosuppressive molecules IL-10 and IFN-γ. We reported the concentration of serum sHLA-G protein as well as mRNA expression levels before and after receiving therapy to establish sHLA-G as diagnostic and prognostic protein marker for HNSCC patients.

## Results

### Demographic characteristics of study groups

The baseline data of both study groups have been provided in Table 1. We noticed higher frequency of male subjects in both groups in comparison to female subjects. Patients having tobacco consumption habit (62.5%) were more prevalent in the study cohort. Patients with oral cavity site, node involvement, later stages of tumor and MDSCC histopath were more.

**Table 1:** Baseline clinical characteristics of the study group.

	Study Group (n = 120) (HNSCC patients)	Control Group (n = 99)
**Gender**		
Male	107	78
Female	13	21
**Age (Years)**		
Mean ± SD	49.01 ± 11.20	42.3 ± 12.40
Range	24–80	20–72
**Habits**		
Smoking + Chewing	75	22
All Habits	40	26
No habits	5	51
**Site**		
Oral cavity	52	—
Oropharynx	43	—
Larynx	19	—
Nasopharynx	6	—
**Node**		
N−	54	—
N+	66	—
**Tumor stage**		
T1 + T2	18	—
T3 + T4	102	—
**Histopath**		
MDSCC	79	—
WDSCC	17	—
SCC(Not defined)	21	—
PDSCC	3	—

SCC: squamous cell carcinoma; WDSCC: well differentiated squamous cell carcinoma; MDSCC: moderately differentiated squamous cell carcinoma; PDSCC: poorly differentiated squamous cell carcinoma.

### Estimation of HLA-G, IL-10 and IFN-γ protein level in the study population

#### By SPR

**Figure 1:**
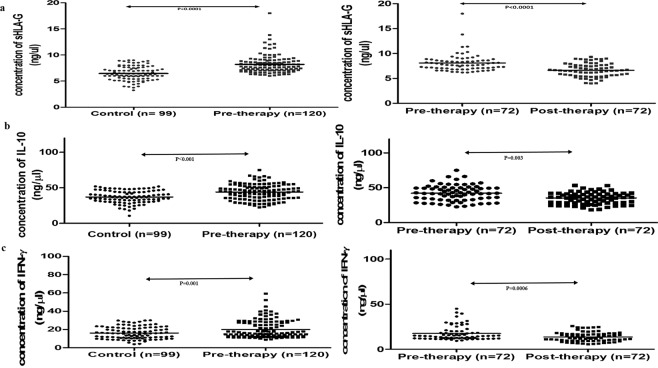
Scatter diagram of mean of proteins concentration in different groups by SPR: (**a**) sHLA-G, (**b**) IL-10, (**c**) IFN-γ.

The standard curves obtained by plotting different concentration of purified proteins and respective RU values are in linear range (Fig. S1). One RU corresponds to immobilized protein concentration of 1 pg/mm2. The RU shows linear relation with concentration of protein which signifies sensitivity of the protein. The protein concentration of all serum samples were estimated using standard curve equation. The concentration of sHLA-G (8.25 ± 1.74 ng/µl), IL-10 (43.99 ± 11.13 ng/µl) and IFN-γ (20.04 ± 9.70 ng/µl) protein levels were higher in serum of HNSCC patients in comparison to controls (sHLA-G: 6.45 ± 1.31; IL-10:37.08 ± 8.55; IFN-γ:16.19 ± 6.60 ng/µl). The obtained range of protein concentration of serum sHLA-G, serum IL-10, and serum IFN-γ in HNSCC patients were 6.04–18 ng/µl, 22.59–75.04 ng/µl and 9.02–59.13 ng/µl, respectively. sHLA-G protein concentration was elevated (8.09 ± 1.79 ng/µl) in patient group which was observed to be declined in concentration levels after therapy (6.64 ± 1.33 ng/µl) which showed response to the therapy on the basis of this protein (Fig. 1). In case of IL-10, the protein concentration was reported 42.43 ± 11.30 ng/µl before therapy and dropped to 34.66 ± 8.33 ng/µl and IFN-γ protein concentration was found 17.45 ± 7.80 ng/µl at the time of diagnosis which declines to 13.62 ± 5.22 ng/µl after completion of therapy (Fig. 1a–c).

**Figure 2:**
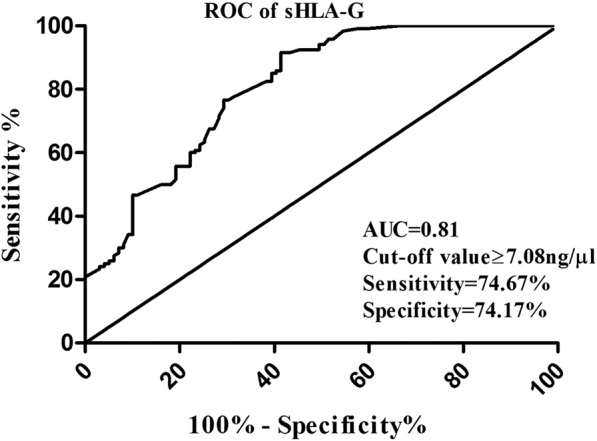
ROC curve analysis: AUC value for sHLA-G to distinguish between HNSCC patients and control.

Receiver-operating characteristic (ROC) curves were prepared to determine diagnostic performance of sHLA-G for HNSCC, where area under curve (AUC) and the cut-off value were 0.81 and ≥7.08 ng/µl with the sensitivity and specificity of 74.67 and 74.17, respectively (Fig. 2).

### sHLA-G protein levels in HNSCC with respect to clinicopathological parameters

To examine the possible role of HLA-G in early diagnosis and staging, we analyzed the sHLA-G protein concentration in serum of HNSCC patients with clinicopathological parameters (Table 2). We found a significant noticeable decline in sHLA-G concentration in T3/T4 stage of tumor (8.16 ± 1.91 to 6.63 ± 1.32), poorly differentiated histopath (8.14 ± 0.62 to 5.65 ± 0.27) after receiving therapy. We have also seen after therapy response in sHLA-G concentration levels in case of male (8.16 ± 1.86 to 6.59 ± 1.31), all age groups (p < 0.0001), smokers (8.05 ± 1.51 to 6.60 ± 1.41), involvement of node (8.62 ± 1.45 to 6.66 ± 1.26), and oropharynx site (7.90 ± 1.24 to 6.10 ± 1.33). Even comparing between patients and controls group, we found that there was a strong association among all age groups, males, and smokers and sHLA-G concentration (p < 0.0001).

**Table 2:** sHLA-G levels estimation by SPR technology in serum of HNSCC patients and the control establishing its correlation with the therapy, represented as mean ± SD.

	Control Group (n = 99) (ng/ul)	Study Group (n = 120) (HNSCC Patients) (ng/ul)	p-value	Study Group (n = 72) (HNSCC patients before therapy) (ng/ul)	Study Group (n = 72) (HNSCC patients after therapy) (ng/ul)	*p*-value**
**Gender**						
Male	6.56 ± 1.21 (78)	8.24 ± 1.74 (107)	**<0.0001**	8.16 ± 1.86 (64)	6.59 ± 1.31 (64)	**<0.0001**
Female	6.10 ± 1.61 (21)*	7.81 ± 1.76 (13)	**0.021**	7.51 ± 1.04 (08)	7.03 ± 1.55 (08)	0.474
**Age (Years)**						
<50 yrs	6.56 ± 1.35 (60)	8.36 ± 1.52 (64)	**<0.0001**	8.05 ± 1.30 (32)	6.68 ± 1.29 (32)	**<0.0001**
>50 yrs	6.42 ± 1.30 (39)	8.01 ± 1.97 (56)	**<0.001**	8.12 ± 2.13 (40)*	6.61 ± 1.40 (40)	**0.0002**
**Habits**						
Smoking + Chewing	6.15 ± 1.13 (22)	8.20 ± 1.56 (75)	**<0.0001**	8.05 ± 1.51 (43)	6.60 ± 1.41 (43)	**<0.0001**
All Habits	6.92 ± 1.37 (26)	8.24 ± 1.90 (40)	**0.030**	8.17 ± 2.96 (26)*	6.62 ± 1.30 (26)	**0.001**
No habits	6.34 ± 1.31 (51)	7.65 ± 0.78 (05)	0.112	7.96 ± 0.77 (03)	7.36 ± 0.72 (03)	0.538
**Site**						
Oral Cavity	—	8.21 ± 1.51 (52)	—	7.79 ± 1.13 (25)	7.22 ± 1.10 (25)	**0.044**
Oropharynx	—	7.94 ± 1.45 (43)	—	7.90 ± 1.24 (27)	6.10 ± 1.33 (27)	**<0.0001**
Larynx	—	8.62 ± 2.54 (19)	—	8.76 ± 2.43 (15)*	6.71 ± 1.52 (15)	**0.01**
Nasopharynx	—	8.54 ± 2.68 (06)	—	8.69 ± 2.41 (05)*	6.46 ± 1.58 (05)	0.222
**Node**						
N−	—	7.73 ± 1.12 (54)	—	7.60 ± 1.04 (37)	6.62 ± 1.42 (37)	**0.0004**
N+	—	8.58 ± 2.06 (66)	—	8.62 ± 1.45 (35)	6.66 ± 1.26 (35)	**<0.0001**
**Tumor stage**						
T1 + T2	—	7.90 ± 1.25 (18)	—	7.70 ± 1.01 (11)	6.72 ± 1.50 (11)	0.0930
T3 + T4	—	7.65 ± 1.82 (102)	—	8.16 ± 1.91 (61)	6.63 ± 1.32 (61)	**<0.0001**
**Histopath**						
MDSCC		8.32 ± 1.91 (79)	—	8.34 ± 2.07 (47)	6.58 ± 1.46 (47)	**<0.0001**
WDSCC		7.93 ± 1.25 (17)	—	7.78 ± 1.20 (07)	7.38 ± 1.34 (07)	0.479
SCC (Not defined)		7.95 ± 1.54 (21)	—	7.44 ± 1.01 (15)	6.68 ± 1.39 (15)	0.051
PDSCC		8.14 ± 0.61 (03)	—	8.14 ± 0.62 (03)	5.65 ± 0.27 (03)	**0.030**

*Non-parametric measures analyzed by Mann–Whitney *U* test **p ≤ 0.05 considered to be significant; N− node absent, N+ node involvement; MDSCC Moderately differentiated squamous cell carcinoma; WDSCC widely differentiated squamous cell carcinoma; SCC squamous cell carcinoma; PDSCC poorly differentiated squamous cell carcinoma.

#### By ELISA

**Figure 3:**
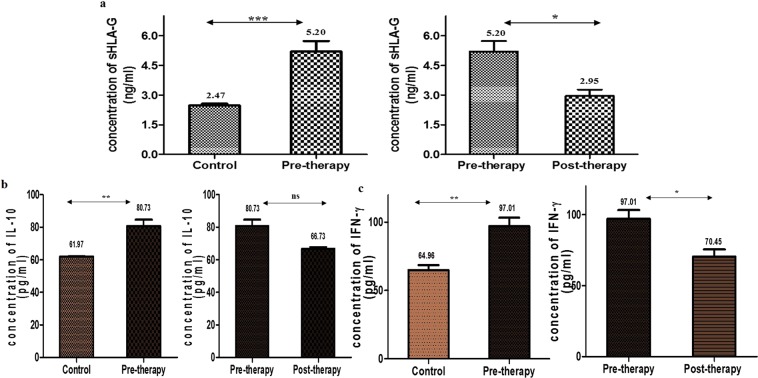
Estimation of proteins in different groups through ELISA (**a**) sHLA-G, (**b**) IL-10, (**c**) IFN-γ.

The concentration of sHLA-G, IL-10 and IFN-γ were also estimated by ELISA by using the respective standard curves (Fig. S1). The concentration of sHLA-G was 2.47 ± 0.10 ng/ml (95% CI:2.22–2.72) for control group, 5.20 ± 0.52 ng/ml (95% CI:3.88–6.51) for pre-therapy patient group and 2.97 ± 0.32 ng/ml (95% CI:2.15–3.75) for post-therapy patient group. The obtained result showed the same pattern as of SPR data. The serum concentrations of IL-10 and IFN-γ were 61.97 ± 0.31 pg/ml (95% CI:61.20–62.73); 64.96 ± 3.59 pg/ml (95% CI:56.04–73.88) for control group, 80.73 ± 6.74 pg/ml (95% CI:63.97–97.49); 97.01 ± 6.27 pg/ml (95% CI:81.54–112.5) for pre-therapy group, and 66.73 ± 1.72 pg/ml (95% CI:62.46–71.00); 70.45 ± 5.0 pg/ml (95% CI:58.04–82.87) for post-therapy group (Fig. 3a–c).

### Quantification of mRNA levels of sHLA-G, IL-10 and IFN-γ by real-time PCR (qRT-PCR)

**Figure 4:**
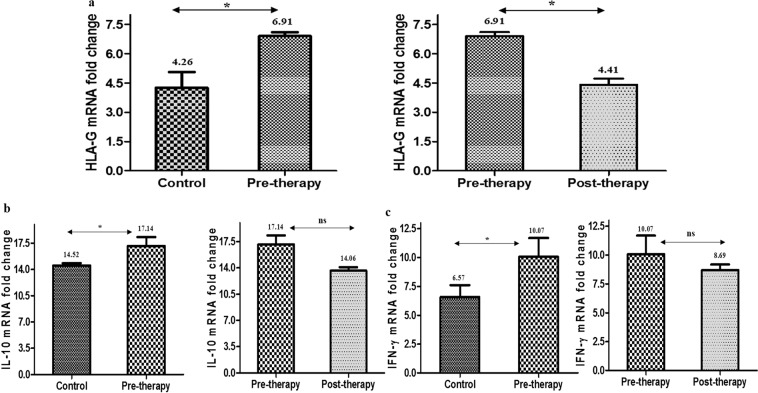
Real time PCR analysis mRNA fold change: (**a**) HLA-G (**b**) IL-10, (**c**) IFN-γ.

The differential expression levels of mRNA of sHLA-G were assessed using quantitative real time PCR in PBMCs of control, pre-therapy and post-therapy groups. The mRNA expression levels were found more than 2 fold higher in comparison to control group (p = 0.032) while post-therapy group showed 1.66 fold lower sHLA-G mRNA levels versus pre-therapy group (p = 0.041). The mRNA levels of IL-10 and IFN-γ were also measured between three groups (Fig. 4a–c).

### Correlation of serum IL-10 and IFN-γ protein concentration with sHLA-G protein

**Figure 5:**
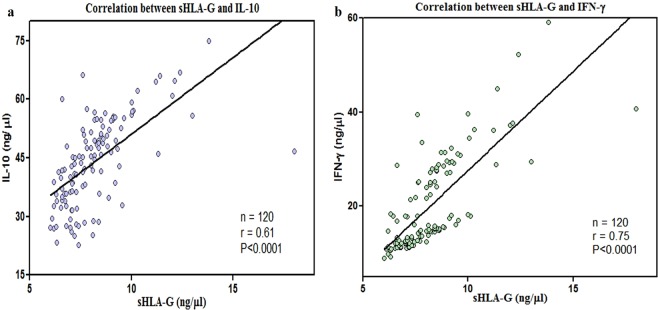
Scatter plot of correlation studies between (**a**) sHLA-G and IL-10 and (**b**) sHLA-G and IFN-γ.

Correlation studies were done among all three proteins using GraphPad Prism 6. The concentrations of proteins were plotted on different axes to obtain a scatter plot. A significant and positive correlation was observed between IFN-γ and sHLA-G (r = 0.75) as well as with IL-10 (r = 0.61) protein concentration in serum of the study group (Fig. 5).

## Discussion

HLA-G molecule has always been an important immunomodulatory role in cancers and associated with tumor immune escape and poor disease prognosis^10^. Differential expression of HLA-G in malignancies has gained clinical interest for developing it as molecular biomarker and therapeutic target^11^. Both forms of HLA-G membrane-bound as well as soluble exert immunomodulatory functions at different stages of the immune response i.e., apoptosis, proliferation and cytokine secretion. HLA-G can have direct inhibition through interaction with ILT2 and ILT4 receptors or prolonged immunosupression through trogocytosis^12^.

The peripheral sHLA-G may exert immunosuppressive functions locally where it’s released as well as at distant sites via blood circulation. Previous studies explored the role of sHLA-G as diagnostic and prognostic markers in various cancers^13–16^. Earlier study found elevated expression of HLA-G and IL-10 in tumour sites and low levels of soluble HLA-G in saliva samples in OSCC^17^. Similarly, another study reported no distinction in salivary concentrations of sHLA-G, IL-10 and TGFβ between oral precancerous lesions and healthy individuals^18^. Shen *et al*., demonstrated association of HLA-G with the prognosis of OSCC using OSCC tissues by IHC and qRT-PCR and may serve as a novel therapeutic target^19^. The Chinese Han population study showed serum levels of sHLA-G in OSCC patients increased significantly with increasing TNM stages^4^. In another report, the plasma sHLA-G levels distinguishing ESCC patients and normal controls by 0.92 AU-ROC value with 70.92% sensitivity^20^. In other cancer research, sHLA-G differentiates healthy controls from breast cancer, colorectal cancer and gastric cancer with AU-ROC of 0.735, 0.97 and 0.91, respectively^16,21^. In a recent study, the expression of sHLA-G levels in the serum and saliva samples of colorectal cancer were analyzed and proposed that sHLA-G could be an attractive molecular target based on its significant high levels in advanced stages^22^. No study has been done so far on serum sHLA-G in HNSCC disease. In present study, significant elevated level of sHLA-G protein (p < 0.0001) was found in serum of HNSCC patients compared to healthy controls and moreover considerably (p < 0.0001) downregulated after the treatment. Same was noted in case of IL-10 and IFN-γ. It has also been observed that sHLA-G protein increased in relation with higher tumor stages and node involvement, oropharynx site which declines at post-therapy. This showed a positive response to therapy in HNSCC patients.

For assessing the potential clinical utility of a biomarker, validation is an unquestionably essential goal. SPR is an optical sensor based method which measures label-free interaction in real time with high sensitivity^23^. In last two decades, it has emerged as a reliable and suitable optical sensor based technique in biomarker validation analyses. This study first time associates sHLA-G serum levels with HNSCC risk using SPR technology. This result was further validated by traditional quantitative ELISA and qRT-PCR experiments. ROC was generated to evaluate the diagnostic performance of sHLA-G and found competently distinguish between two diagnostic groups- control and HNSCC patients with high sensitivity and specificity. The AUC value 0.81 obtained from ROC analysis in the present study with 74.67% sensitivity and 74.17% specificity can provide sHLA-G to be a potential diagnostic protein marker to distinguish HNSCC from healthy control and it is explored for the first time, establishing its correlation with the therapy response.

Been highly aggressive in nature, HNSCC tumors have been involved in different mechanisms to evade immune recognition such as downregulation or loss of human leukocyte antigen (HLA) class I molecules, and/or disruption of the antigen-processing machinery (APM), expression of the non-classical human leukocyte antigen HLA-G, known to inhibit natural killer (NK) cells, T cells and antigen-presenting cells (APC), release of immunosuppressive factors into the tumour microenvironment e.g. IL-10, IL-6, transforming growth factor-β^2,24–26^. Immune responses in HNSCC are associated with a shift from Th1 (IFN-γ, IL-2) to Th2 (IL-4, IL-6 and IL-10) cytokine production^27^. In this scenario, cytokines effect on immunosuppression ability of HLA-G by regulating its expression levels. HLA-G has been also shown to modulate the release of cytokines from peripheral blood mononuclear cells or get modulated by several cytokines such as IL-10 and IFNs^28^. In accordance with this, a study from north-east suggests that IFN-γ expression appeared to be mediated by HLA-G in HNSCC tissues and through regulating HLA-G expression, HPV positive tumors could mediate immune suppression by manipulating SOCS, IFN-γ, IL-10 and cyclin D1 pathways^29^. In view of previous study, we have correlated the proinflammatory (IFN- γ) and anti-inflammatory cytokines (IL-10) with of sHLA-G levels in HNSCC patients. Correlation studies showed a positive relation between sHLA-G, IL-10 and IFN-γ which supports the outcome of previous literature.

Taken together, present study attempts to evaluate the sHLA-G levels in serum of HNSCC patients before therapy and after therapy and correlated with clinicopathological parameters. Further, the correlation of IL-10 and IFN-γ protein levels in serum supported the cytokines mediate effect on expression levels of serum sHLA-G protein. Our findings reveal that the sHLA-G could be a potential diagnostic as well as prognostic serum protein marker with its clinical utility to monitor the response of therapy.

## Materials and Methods

### Study groups

We enrolled 120 HNSCC patients and 99 ethically matched healthy controls in this retrospective study. The blood samples were collected from Head and Neck Cancer Clinic, Dr. B.R.A. Institute Rotary cancer Hospital (IRCH), All India Institute of Medical Sciences (AIIMS), New Delhi, India from 2016 to 2018. This study was approved by All India Institute of Medical Sciences (AIIMS) ethics sub-committee (IESC/T-469.12.2014). All methods were performed in accordance with the relevant ethical guidelines and regulations. Patients with histologically proven squamous cell carcinoma having primary sites from oral cavity, oropharynx, larynx and nasopharynx were recruited. Patients with any serious illness, chronic infection, inflammatory diseases, and any history of cancer were excluded from the study. The staging was done in the TNM classification according to the 7^th^ edition of American Joint Committee on Cancer (AJCC).Written informed consent forms were obtained from both the groups.

### Treatment

The majority of patients presented in the clinic at their advanced stages so combined modality treatment (CMT) was preferred which comprises of surgery followed by post-operative radio-therapy or concurrent chemo-radiotherapy depending on patient’s age, tumor stage, performance status and preference. Radiotherapy dose usually consists of 2 Gy per fraction, delivered for five days in a week for a total duration of 6–7 weeks on Co60 or linear accelerator. Cisplatin was used commonly for the treatment of scheduled dose 40 mg/m^2^ administered in 5 cycles, 1 cycles/week. Present study evaluated serum levels of sHLA-G, IL-10 and IFN-γ proteins at the time of diagnosis (pre-therapy), and 2 months after receiving treatment (post-therapy). Blood samples of 120 patients were recruited at pre-therapy and only 72 patients attended after 2 months of treatment for follow-up as 41 patients dropped out of the study, 2 patients died and 5 patients were sent to supportive care clinic.

### Sample collection and preparation

Blood samples (5 ml) were withdrawn 2 times during the study: 1- at the time of diagnosis (pre-therapy), 2- after receiving therapy (post-therapy such as surgery, chemotherapy or radiotherapy). Blood samples (2 ml) were allowed to clot at room temp for 45 min and centrifuged at 3,000 rpm for 5 min. Buffy coat was removed and serum was collected in microcentrifuge tubes (MCT) and kept at −80 °C until use.

Three milliliters of blood sample was taken in heparin coated vacutainers for real time quantitative polymerase chain reaction (RT-qPCR) analysis. After withdrawing blood, vials were kept on rocker for 30 min to maintain the room temperature, PBMC were isolated using Histopaque (Sigma-Aldrich, USA) density gradient centrifugation method following manufacturer’s instructions. Isolated PBMCs were used for total RNA isolation through Trizol method using RiboZol RNA extraction reagent (Amresco, USA). The purity, integrity, and quantification of the RNA samples were analyzed using the Bioanalyser (Agilent Technologies, USA).

### Estimation of sHLA-G, IL-10 and IFN-γ proteins level in the study population

#### By surface plasmon resonance (SPR)

SPR, an optical biosensor based system which is best utilized for real time specific interaction analysis, was used for the estimation of HLA-G, IL-10 and IFN-γ proteins concentration level in the serum of study groups. All SPR measurements were performed using the BIAcore- 3000 apparatus (Wipro GE Healthcare, UK) at 25 °C. The anti-HLA-G monoclonal antibody MEM-G/9 (sc-51678; Santa Cruz Biotech Inc., U.S.A.), anti-IL-10 monoclonal antibody (sc-8438, Santa Cruz Biotech Inc., U.S.A.) and anti-IFN-γ monoclonal antibody (sc-390800; Santa Cruz Biotech Inc., U.S.A.) antibodies were immobilized on three different flow cells of CM5 sensor chip using the amine coupling kit (Wipro GE Healthcare, UK). Equilibration of system was done using HBS-EP (Wipro GE Healthcare, UK) as a running buffer with a maintained flow rate of 5 μl/min. The standard curves were prepared by passing different concentrations of purified recombinant HLA-G protein (0.931, 2.793, 4.655, 9.31, 13.965, and 18.62 ng/μl), IL-10 protein (7.15, 14.3, 28.6, 42.9 and 57.2 ng/μl) and IFN- γ (4.29, 7.15, 14.3, 28.6, 42.9 and 57.2 ng/μl) protein over their respective immobilized antibodies. All proteins were diluted with HBS-EP buffer before running on the sensor chip. The generated SPR signal was measured as Response Units (RU) and used for standard curve preparation. In a similar way, serum of study groups were passed over immobilized antibodies and RU was recorded. Protein concentration of serum samples of all study groups were derived using their respective standard curves.

#### By enzyme-linked immunosorbent assay (ELISA)

Sandwich ELISA was performed for further validation of data obtained from SPR. The proteins sHLA-G, IL-10 and IFN-γ were further measured using the commercial human Major Histocompatibility Complex Class I G (MHCG) (SEB856Hu), human IL-10 (SEA056Hu), and human IFN-γ (SEA049Hu) ELISA kits (Cloud Clone Corp., Houston, U.S.A.). Experiments were performed as per manufacturers’ instructions. The precoated biotin-conjugated monoclonal anti‐human sHLA‐G antibody specific to MHCG, was used as a capture antibody and an anti‐β2‐microglobulin antibody conjugated to Horseradish Peroxidase (HRP) was used as the secondary antibody. TMB substrate solution was added which exhibited a change in color on the presence of MHCG binding with antibodies. The enzyme-substrate reaction was terminated by adding sulphuric acid solution and the color change was measured spectrophotometrically at a wavelength of 450 nm.The minimum detectable dose of sHLA-G, IL-10 and IFN-γ were <0.17 ng/ml, 3 pg/ml and 5.9 pg/ml, respectively. Serum samples were assayed in triplicate with 1:5 dilution of serum with 1X Phosphate Buffer Saline (PBS) buffer. The sHLA-G protein concentrations used for standard curve preparation were 24, 12, 6, 3, 1.5, 0.75 and 0.38 ng/ml. The protein concentrations used for standard curve preparation for IL-10 and IFN-γ were 500, 250, 125, 62.5, 31.2, 15.6 and 7.8 pg/ml, and 1000, 500, 250, 125, 62.5, 31.2, and 15.6 pg/ml, respectively.

### Quantification of mRNA level of HLA-G, IL-10 and IFN-γ by real-time PCR (qRT-PCR)

Total RNA was isolated from PBMCs using RiboZol RNA extraction reagent (Amresco, USA) as per manufacturer’s instructions. Briefly, 1 ml of RiboZol (a mixture of guanidine thiocyanate and phenol) per 5×10^6^ cells was added directly in MCT and passed several times through a pipette to lyse the cells. Thereafter, 0.2 ml of chloroform per 1 ml of RiboZol reagent was added in the homogenized sample and centrifuged. The aqueous phase exclusively contains RNA, was transferred to a fresh tube. RNA was precipitated by adding 0.5 ml of isopropyl alcohol. RNA precipitate looked like a gel-like pellet which was further washed with 75% ethanol. The RNA pellet was air-dried and dissolved in RNAse free water/DEPC-treated water. The integrity and quality was checked by 2% denaturing agarose gel electrophoresis and concentration was determined using nanodrop instrument (BioTek Instruments, Inc., U.S.A.). RNA samples exhibited intact 28 S and 18 S bands were used for further experiments. RNA samples were kept at −80 °C for long term storage.

One microgram of total RNA was reverse transcribed into cDNA using RevertAid First Strand cDNA Synthesis Kit (Thermofisher, USA) as per protocol provided by manufacturer. Briefly, 1 µg of total RNA was mixed with 1 µl of random hexamer primer and volume was made upto 12.5 µl. Mixture was centrifuged and incubated at 75 °C for 5 min, chill on ice for 1 min. The following components were added: 5X reaction buffer- 4 µl, RiboLockRNAse Inhibitor- 0.5 µl, dNTP Mix- 2 µl, Reverse Transcriptase- 1 µl, and incubated at 25 °C for 10 min followed by 60 min at 37 °C. All incubation steps were done using T100 thermal cycler (Bio-Rad Laboratories, Inc., U.S.A.). β-actin was used as an internal control. One microgram of cDNA was added to detect the amplification of specific PCR products of sHLA-G and β-actin in Brilliant III Ultra-Fast SYBR® Green QPCR Master Mix (Agilent Technologies, Inc., U.S.A.) using Mx3005P QPCR system (Agilent Technologies, Inc., U.S.A.). Total reaction mixture (20 µl) was as follows: QPCR Master Mix (10 µl), forward primer (1 µl of 10 µM), reverse primer (1 µl of 10 µM), cDNA (1 µg), and nuclease-free water (as required). The reactions were performed in triplicates. Primers were designed using IDT PrimerQuest tool (Integrated DNA Technologies, Inc., U.S.A.) and obtained from GCC BIOTECH (INDIA) PVT.LTD. Primers used for these genes were: HLA-G forward primer- 5′ATCATGGGTATCGTTGCT 3′, HLA-G reverse primer- 5′CTCCCTCCTTTTCAATCTG 3′; IL-10 forward primer- 5′GCTGGAGGACTTTAAGGGTTAC 3′, IL-10 reverse 5′GATGTCTGGGTCTTGGTTCTC 3′; IFN-γ forward- 5′TTCTCTTGGCTGTTACTG 3′, IFN-γ reverse 5′GATGTAGCGGATAATGGAAC 3′; β-actin Forward primer, 5′TGGCACCCAGCACAATGAA 3′, Reverse primer, 5′CTAAGTCATAGTCCGCCTAGAAGCA 3′. The reaction conditions consisted of one cycle of 10 min at 95 °C, 40 cycles of 30 s at 45 °C (HLA-G), at 63 °C (IL-10), at 49 °C (IFN-γ) and 1 min at 60 °C, and ended up with a melting curve analysis.

### Statistical analysis

Statistical analysis was carried out using Graph Pad Prism version 6.0 (California corporation). Unpaired t-test was performed on two different variables and paired t-test was done on two paired variables. By using rule of thumb, given by Altmann^30^, we analyzed normality of all parameters. As few parameters did not follow a Gaussian distribution, the differences of sHLA-G concentration between groups were analyzed using the Mann–Whitney *U* test. ROC curves were prepared to identify cutoff value for all three proteins. The p-value <0.05 was considered statistically significant.

### Ethical approval

This study was approved by All India Institute of Medical Sciences (AIIMS), New Delhi, India ethics sub-committee (IESC/T-469.12.2014).

### Informed consent

Informed consent was obtained from all individual participants included in the study.

## Supplementary information

Supplementary figure.

## References

[1] González Ã (2012). The immunosuppressive molecule HLA-G and its clinical implications. Crit. Rev. Clin. Laboratory Sci..

[2] Duray A, Demoulin S, Hubert P, Delvenne P, Saussez S (2010). Immune Suppression in Head and Neck. Cancers: A Review. Clin. Developmental Immunology.

[3] Lin A, Yan W-H (2015). Human Leukocyte Antigen-G (HLA-G) Expression in Cancers: Roles in Immune Evasion, Metastasis and Target for Therapy. Mol. Med..

[4] Wang Z, Zhao L, Liu L, Liu X (2018). Human leucocyte antigen-G 14-bp InDel polymorphism and oral squamous cell carcinoma risk in Chinese Han population: A case-control study. Int. J. Immunogenet..

[5] Urosevic M, Dummer R (2003). HLA-G and IL-10 expression in human cancer–different stories with the same message. Semin. Cancer Biol..

[6] Rodríguez JA (2012). Altered HLA Class I and HLA-G Expression Is Associated with IL-10 Expression in Patients with Cervical Cancer. Pathobiology.

[7] Ibrahim EC (2001). Tumor-specific up-regulation of the nonclassical class I HLA-G antigen expression in renal carcinoma. Cancer Res..

[8] Castelli EC (2009). In silico analysis of microRNAS targeting the HLA-G 3′ untranslated region alleles and haplotypes. Hum. Immunology.

[9] Agnihotri V (2017). Promising link of HLA-G polymorphism, tobacco consumption and risk of Head and Neck Squamous Cell Carcinoma (HNSCC) in North Indian population. Hum. Immunology.

[10] Yie S, Hu Z (2011). Human leukocyte antigen-G (HLA-G) as a marker for diagnosis, prognosis and tumor immune escape in human malignancies. Histol. Histopathol..

[11] Zhang Y, Yu S, Han Y, Wang Y, Sun Y (2017). Human leukocyte antigen-G expression and polymorphisms promote cancer development and guide cancer diagnosis/treatment (Review). Oncol. Lett..

[12] Curigliano G, Criscitiello C, Gelao L, Goldhirsch A (2013). Molecular Pathways: Human Leukocyte Antigen G (HLA-G). Clin. Cancer Res..

[13] Attia MA, Nosair NA, Gawally A, Elnagar G, Elshafey EM (2014). HLA-G Expression as a Prognostic Indicator in B-Cell Chronic Lymphocytic Leukemia. Acta Haematol..

[14] Chen H-X (2010). Upregulation of human leukocyte antigen–G expression and its clinical significance in ductal breast cancer. Hum. Immunology.

[15] Farjadian, S. *et al*. HLA-G Expression in Tumor Tissues and Soluble HLA-G Plasma Levels in Patients with Gastrointestinal Cancer. *Asian Pac J Cancer Prev* **19** (2018). 10.22034/APJCP.2018.19.10.2731 PMC6291033 30360598

[16] Cao M (2011). Plasma soluble HLA-G is a potential biomarker for diagnosis of colorectal, gastric, esophageal and lung cancer. Tissue Antigens.

[17] Gonçalves AS (2015). Immunosuppressive mediators of oral squamous cell carcinoma in tumour samples and saliva. Hum. Immunol..

[18] Gonçalves AS (2017). Overexpression of immunomodulatory mediators in oral precancerous lesions. Hum. Immunology.

[19] Shen X (2018). Correlation between human leukocyte antigen-G expression and clinical parameters in oral squamous cell carcinoma. Indian. J. Cancer.

[20] Zheng J (2014). Human leukocyte antigen G is associated with esophageal squamous cell carcinoma progression and poor prognosis. Immunology Lett..

[21] Provatopoulou X (2012). Soluble human leukocyte antigen-G expression in patients with ductal and lobular breast malignancy. Anticancer. Res..

[22] Lázaro-Sánchez, A. D. *et al*. HLA-G as a new tumor biomarker: detection of soluble isoforms of HLA-G in the serum and saliva of patients with colorectal cancer. *Clin Transl Oncol*, 10.1007/s12094-019-02244-2 (2019). 10.1007/s12094-019-02244-2 31748960

[23] Köhler K, Seitz H (2012). Validation Processes of Protein Biomarkers in Serum—A Cross Platform Comparison. Sens..

[24] Meissner M (2005). Defects in the Human Leukocyte Antigen Class I Antigen Processing Machinery in Head and Neck Squamous Cell Carcinoma: Association with Clinical Outcome. Clin. Cancer Res..

[25] Silva TG (2011). Expression of the nonclassical HLA-G and HLA-E molecules in laryngeal lesions as biomarkers of tumor invasiveness. Histol. Histopathol..

[26] Allen CT, Judd NP, Bui JD, Uppaluri R (2012). The clinical implications of antitumor immunity in head and neck cancer. Laryngoscope.

[27] Bussu F (2018). IFN-γ and other serum cytokines in head and neck squamous cell carcinomas. Acta Otorhinolaryngol. Ital..

[28] Carosella, E. D. *et al*. HLA-G Molecules: from Maternal–Fetal Tolerance to Tissue Acceptance. *Advances in Immunology* **81** 199–252 (Elsevier, 2003). 10.1016/s0065-2776(03)81006-4 14711057

[29] Sarmah N, Baruah MN, Baruah S (2019). Immune Modulation in HLA-G Expressing Head and Neck Squamous Cell Carcinoma in Relation to Human Papilloma Virus Positivity: A Study From Northeast India. Front. Oncol..

[30] Altman DG, Bland JM (1996). Statistics Notes: Detecting skewness from summary information. BMJ.

