# Supplementary material for: Serum sHLA-G: Significant diagnostic biomarker with respect to therapy and immunosuppressive mediators in Head and Neck Squamous Cell Carcinoma
Source: Sci Rep. 2020 Mar 2;10:3806. doi: 10.1038/s41598-020-60811-y (PMC7052243; doi:10.1038/s41598-020-60811-y)
Supplement: Supplementary file 1 — Supplementary figure. [file 41598_2020_60811_MOESM1_ESM.pdf]

Title: Serum sHLA-G: Significant diagnostic biomarker with respect to therapy and immunosuppressive mediators in Head and Neck Squamous Cell Carcinoma

Vertica Agnihotri, Abhishek Gupta, Lalit Kumar and Sharmistha Dey*

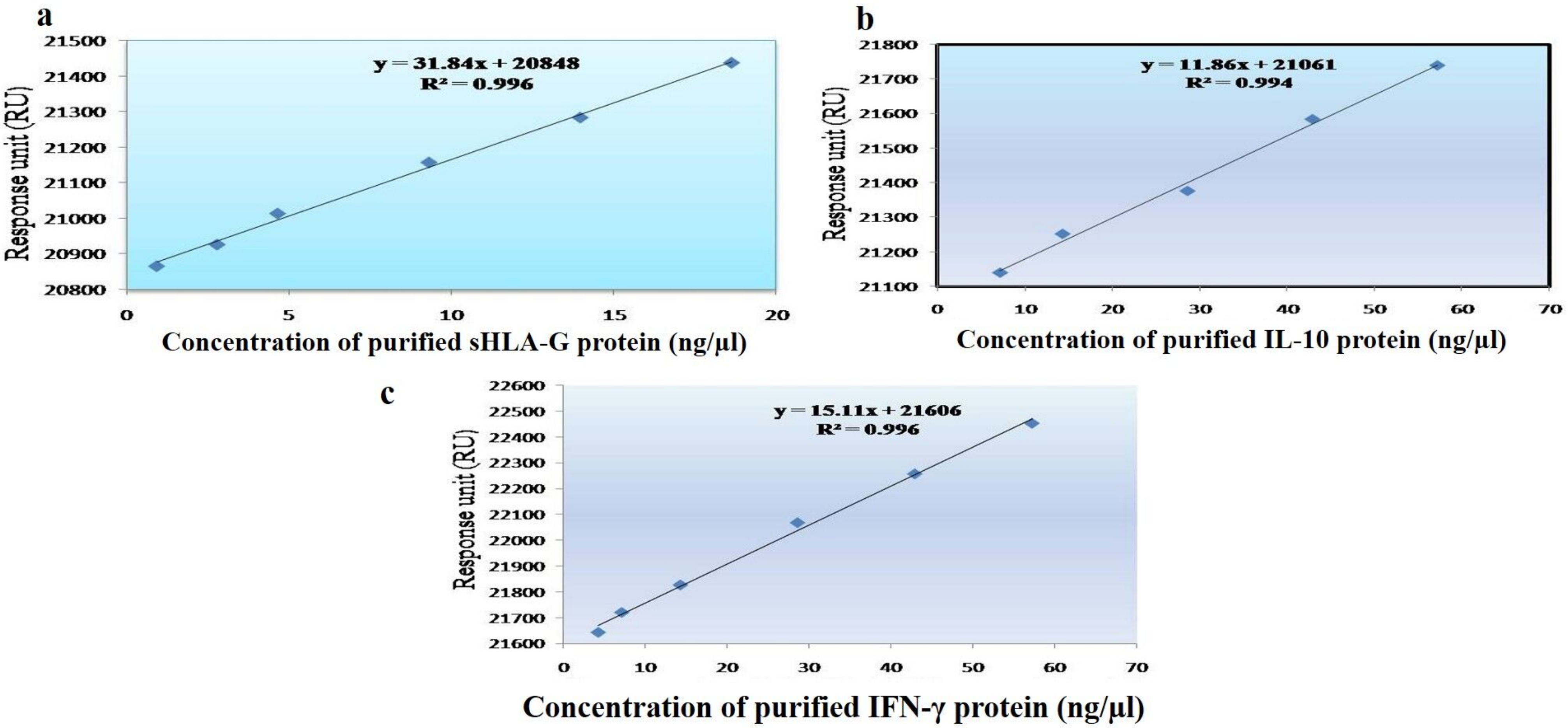

Figure S1. SPR: Standard curves between response units and concentration of protein

Title: **Serum sHLA-G: Significant diagnostic biomarker with respect to therapy and immunosuppressive mediators in Head and Neck Squamous Cell Carcinoma**

Vertica Agnihotri, Abhishek Gupta, Lalit Kumar and Sharmistha Dey*

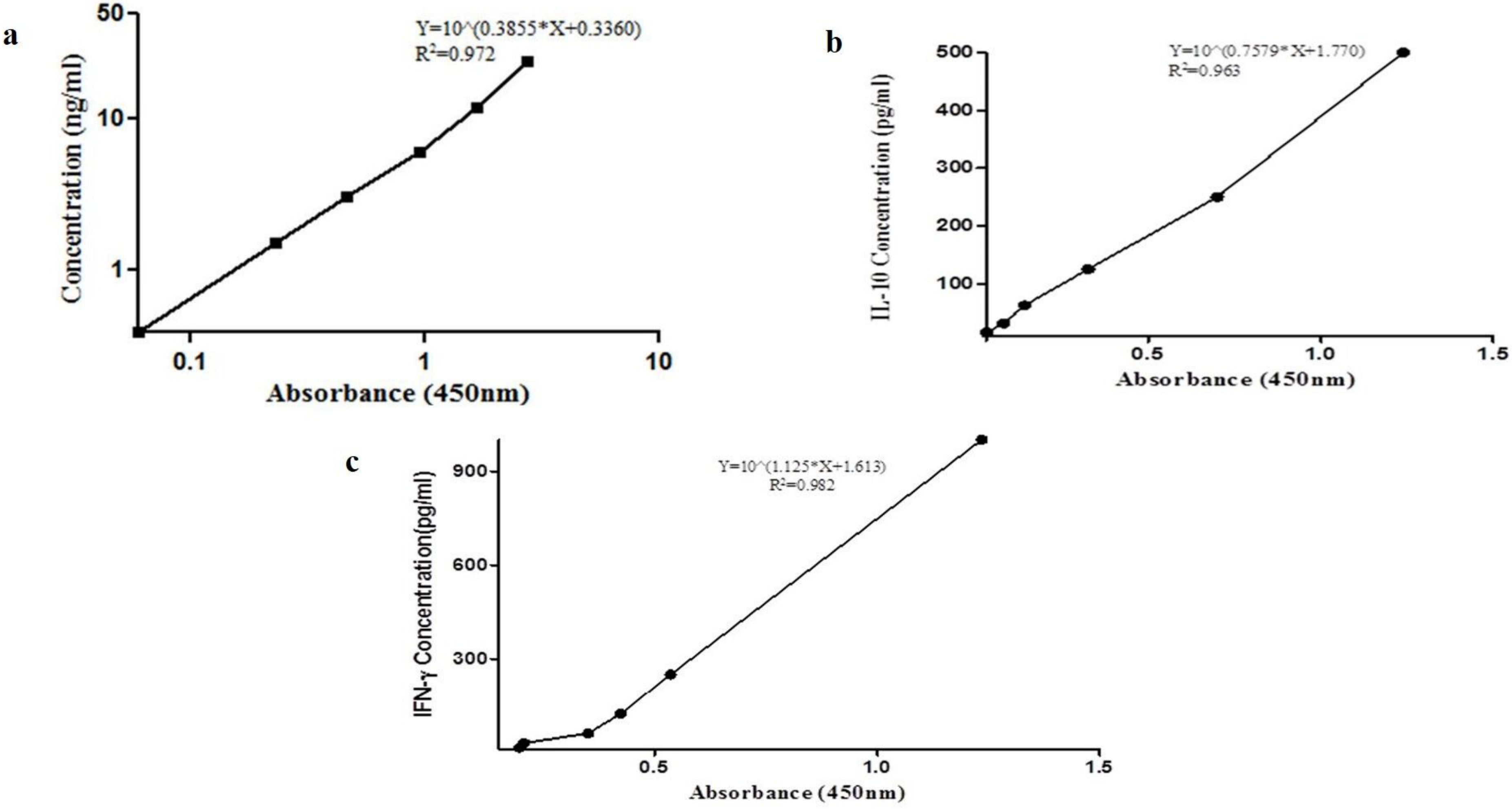

Figure S2. ELISA: Standard curves between absorbance unit and concentration of proteins, (a) sHLA-G, (b) IL-10, (c) IFN-γ
